# Utilization and Sustainability Evaluation of Steel Slag and RAP in Hot Recycled Asphalt Mixtures—Case Study

**DOI:** 10.3390/ma19061231

**Published:** 2026-03-20

**Authors:** Liang Song, Zijie Xie, Jie Gao, Chong Gao, Le Wang, Mingwen Tao

**Affiliations:** 1 School of Traffic and Transportation Engineering, Xinjiang University, Urumqi 830017, China; 2 Xinjiang Uygur Autonomous Region Highway Development Center, Urumqi 830000, China; 3 College of Ecology and Environment, Xinjiang University, Urumqi 830017, China; 4 School of Civil Engineering and Architecture, East China Jiaotong University, Nanchang 330013, China; 5 Xinjiang Transportation Investment Construction Management Co., Ltd., Urumqi 830099, China; 6 School of Transportation and Logistics, Xinjiang Agricultural University, Urumqi 830091, China

**Keywords:** steel slag, RAP, environmental benefit, economic performance, transport distance

## Abstract

To address natural aggregate scarcity and improve the high-value utilization of Reclaimed Asphalt Pavement (RAP), this study proposes a steel slag–RAP hot recycled asphalt mixture (SSRM) as a sustainable alternative to conventional limestone–RAP mixtures (RM). Unlike previous studies mainly focusing on performance verification, an integrated environmental–economic evaluation framework was developed based on real highway expansion project data under a “cradle-to-gate” boundary and incorporating transportation distance thresholds. SSRM containing 50% RAP and 23% steel slag as coarse aggregate replacement was evaluated through rutting, semi-circular bending (SCB), freeze–thaw splitting (TSR), and skid resistance tests. Compared with RM, SSRM exhibited 14–16% higher dynamic stability and 20–25% higher fracture energy at −10 °C, along with improved moisture stability and skid resistance, mainly attributed to the rough and alkaline characteristics of steel slag enhancing adhesion and aggregate interlocking. Life-cycle assessment (GWP100) and cost analysis indicate that SSRM reduces carbon emissions by 10–11% relative to RM and about 40% compared with conventional virgin mixtures, while initial construction costs decrease by 9–10%. Transportation sensitivity analysis identifies equal-emission and equal-cost thresholds of approximately 590 km and 380 km, respectively. Within typical material supply radii (300–400 km), SSRM demonstrates both environmental and economic advantages, providing a practical framework for low-carbon material selection in highway construction.

## 1. Introduction

In recent years, the continuous expansion of transportation infrastructure has generated an immense demand for natural aggregates. It is estimated that the global consumption of construction aggregates exceeds 50 billion tons annually, making it the most extracted solid material worldwide. This growing demand has led to severe resource depletion and significant carbon emissions associated with construction materials [1]. This has necessitated a shift in road engineering toward green and low-carbon development. Utilizing solid waste as a substitute for natural aggregates has emerged as an effective pathway to achieve sustainable development [1,2,3]. Currently, two technical approaches have reached relative maturity: the plant-mixed hot recycling of Reclaimed Asphalt Pavement (RAP) and the utilization of steel slag as a substitute for natural aggregates in asphalt mixture production. However, when either technology is applied individually, the incorporation ratio of solid waste is often constrained by mixture design specifications and the need to balance mechanical performance. These limitations create a bottleneck in the overall capacity for waste consumption. Consequently, the synergistic replacement of natural aggregates with both steel slag and RAP not only overcomes the limitations of single-waste utilization but also substantially increases the total solid waste content in recycled asphalt mixtures. In conventional hot recycling practice, the RAP content is typically limited to about 20–30% due to performance constraints. By incorporating steel slag aggregates simultaneously, the total solid waste substitution ratio can potentially increase to approximately 50–70% of the aggregate mass, thereby achieving a higher level of resource recovery.

In response, the academic community has initiated explorations into the synergistic application of steel slag and RAP materials within a unified mixture system. First, regarding the macro-mechanical performance and mix proportion optimization of the mixture, multiple studies have confirmed the feasibility of their synergy. Georgiou and Loizos [4] employed electric arc furnace steel slag in combination with 25–50% high-content RAP to design a warm-mix asphalt mixture as an alternative to conventional surface layers. Their research indicated that the combination of steel slag and RAP generally outperformed the control group in terms of stiffness, rutting resistance, and cracking resistance, while also meeting specifications for moisture stability. Fakhri and Ahmadi [5] further compared different blending ratios within a warm-mix system and identified that a combination of approximately 40% steel slag coarse aggregate and 40% RAP fine aggregate yielded optimal performance in rutting resistance, cracking resistance, and elastic modulus, suggesting a potentially favorable interaction between steel slag and RAP under the investigated mixture conditions. However, the findings reported in the literature are not fully comparable, because the studies differ substantially in RAP content, steel slag source and gradation, asphalt binder system, production temperature, and test protocols. As a result, although the feasibility of combining steel slag and RAP has been widely indicated, the extent of the reported performance improvement remains study-dependent. Second, concerning the enhancement of functional properties of the mixture, researchers have explored the multi-scale application potential of steel slag. The quantitative evaluation by Wang et al. [6] demonstrated that, owing to the rich surface texture of steel slag, high-content RAP–steel slag systems exhibited significantly superior macro-texture depth and pendulum friction coefficients compared to basalt control groups, confirming their complementary advantages in skid resistance. From a micro-filler perspective, Naser et al. [7] found that incorporating steel slag powder as filler in a 30% RAP recycled mixture significantly improved dynamic stability and fatigue life, indicating that steel slag contributes to enhanced pavement performance not only as coarse aggregate but also at finer scales. Additionally, Song et al. [8] achieved comprehensive improvements in high-temperature performance, moisture stability, and fatigue-cracking resistance of steel slag–RAP systems by introducing epoxy asphalt, thereby offering a novel pathway for high-performance recycling technologies. Finally, building upon pavement performance research, some scholars have extended their perspective to full life-cycle environmental benefit assessments. Based on previous studies, Georgiou et al. [9] established a cradle-to-gate life cycle assessment model, revealing that the scenario employing 50% RAP with steel slag replacing natural aggregate exhibited the lowest greenhouse gas emissions (GWP100) during the raw material acquisition and mixing stages. Supporting case studies [10,11] further corroborated that the combined use of steel slag and high-content RAP significantly reduces the consumption of virgin aggregates and base asphalt, representing a key technological pathway for constructing pavement systems with both high performance and high recycling rates. Nevertheless, most of the above studies primarily focus on laboratory-scale performance evaluation, while systematic environmental and economic assessments remain relatively limited. In addition, several studies were conducted within warm-mix asphalt (WMA) systems, which operate at lower production temperatures than conventional plant-mixed hot recycling processes. Because production temperature may influence binder aging characteristics, mixture compaction behavior, and mixture performance, the applicability of these findings to plant-mixed hot recycled asphalt mixtures still requires further investigation. Therefore, further investigation under plant-mixed hot recycling conditions is necessary to clarify the engineering applicability of steel slag–RAP mixtures.

Despite the growing academic interest in the combined use of steel slag and RAP in asphalt mixtures, several issues remain insufficiently addressed. Existing studies have mainly emphasized mixture design and engineering performance, whereas integrated assessments of environmental impact and economic feasibility are still relatively limited [11]. Moreover, many published evaluations are based on laboratory scenarios or simplified life-cycle boundaries, with less attention given to real project conditions such as material sourcing and transportation distance. Therefore, further project-based analysis is needed to clarify the practical sustainability implications of applying steel slag–RAP recycled mixtures in highway engineering.

**Figure 1:**
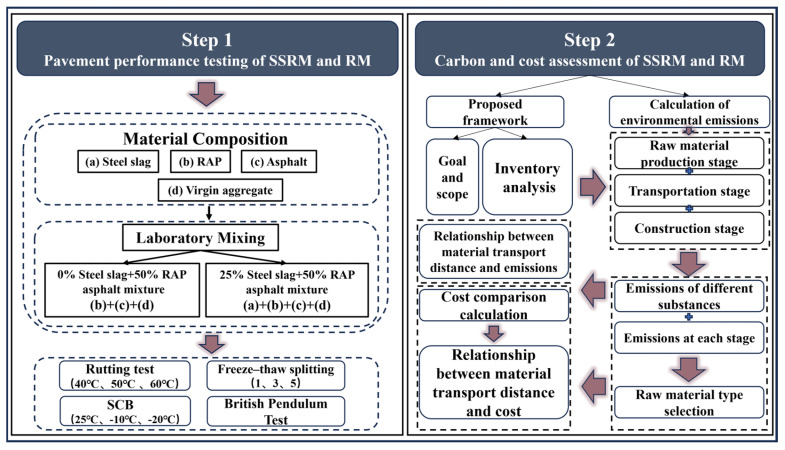
Research flow chart.

Responding to this identified research gap, this study leverages the G30 Lianyungang-Khorgos Expressway expansion project in Xinjiang, China, as a real-world engineering backdrop. Specifically focusing on the mid-surface layer of steel slag-RAP plant-mixed hot recycled asphalt mixture, this research aims to provide a comparable and quantifiable basis for its engineering application in heavy-load expressways within arid Northwestern regions, across performance, environmental, and economic dimensions. To achieve this, the study undertakes a two-pronged approach: First, it evaluates the performance of Steel Slag-RAP Recycled Mixture against Limestone-RAP Recycled Mixture through laboratory tests, including rutting, low-temperature cracking, freeze–thaw splitting (TSR), and skid resistance. This comparative analysis will assess the engineering feasibility and performance advantages of replacing natural aggregates with steel slag in recycled mixtures. Secondly, within the same functional unit and system boundary, the study constructs an environmental emission calculation model and a one-time construction cost model. By identifying equal emission and equal cost thresholds that vary with transportation distance, this research will provide crucial insights into the environmental and economic trade-offs associated with different material choices. The overall research framework is illustrated in Figure 1.

Therefore, the primary objectives of this study are:

- To compare the road-performances of steel slag–RAP recycled asphalt mixture and limestone–RAP recycled asphalt mixture, thereby evaluating the engineering feasibility and performance advantages of substituting natural aggregates with steel slag.
- To construct an engineering scenario-specific environmental emission calculation model and a one-time construction cost evaluation model, incorporating key parameters such as transportation distance, identify equal emission and equal cost thresholds along with their applicable boundaries.

## 2. Materials and Methods

### 2.1. Raw Materials

(1) Steel slag

The steel slag was collected from Xinjiang Bayi Iron & Steel Co., Ltd. (Urumqi, China), and the basic properties are presented in Table 1.

**Figure 2:**
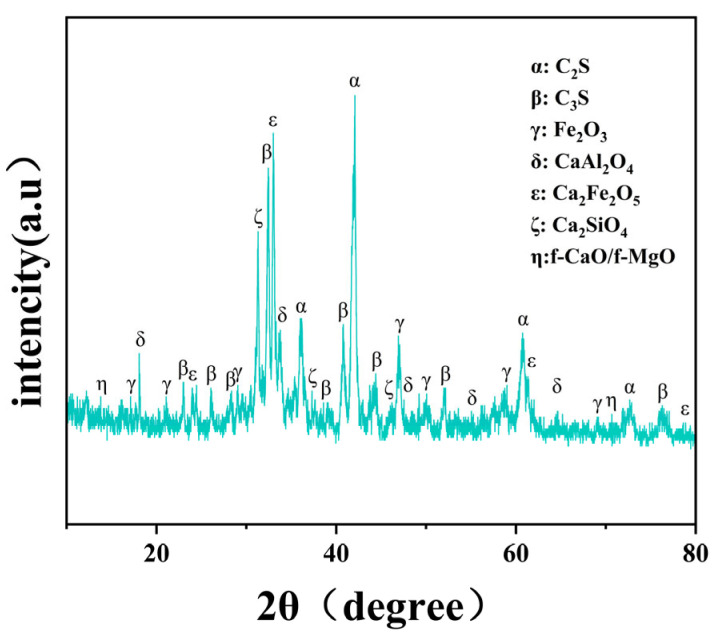
XRD Spectrum of Steel Slag Powder.

To investigate the mineral composition and microstructure of steel slag, XRD analysis was performed on steel slag samples after drying, grinding, and passing through a 200-mesh sieve. The tests were conducted using CuKα radiation (λ = 1.5406 Å), with a scanning range of 5–80° (2θ) and a step size of 0.02°. As shown in Figure 2, the XRD spectrum of steel slag revealed its main crystalline phases consist of calcium silicates (Ca_2_SiO_4_, C_2_S), calcium aluminates (Ca_3_Al_2_O_6_, C_3_A), and iron oxides (FeO, Fe_2_O_3_), accompanied by small amounts of calcium hydroxide (Ca (OH)_2_) and free calcium oxide (f-CaO). The steel slag was obtained from Xinjiang Bayi Iron & Steel Co., Ltd. and naturally aged for more than six months according to (JTG F40–2004) [12], which promotes the hydration of free CaO and MgO and improves volumetric stability.

(2) Limestone coarse aggregates

The used limestone was collected from a crushing plant in south Xinjiang; its technical properties meet the requirements of the “Test Methods of Aggregates for Highway Engineering” (JTG 3432-2024) [13], as presented in Table 2.

(3) Reclaimed Asphalt Pavement (RAP)

The reclaimed asphalt pavement (RAP) was generated by the G30 expansion project in Xinjiang, which has been serviced for over 20 years. Its asphalt content is 4.12%, and the gradation is shown in Table 3.

(4) Asphalt

The asphalt used in this study was 70# road petroleum asphalt; its properties met the requirements of the “Standard Test Methods of Bitumen and Bituminous Mixtures for Highway Engineering” (JTG E20-2011) [14], as presented in Table 4.

### 2.2. Asphalt Mixture Design and Pavement Performance Evaluation

**Figure 3:**
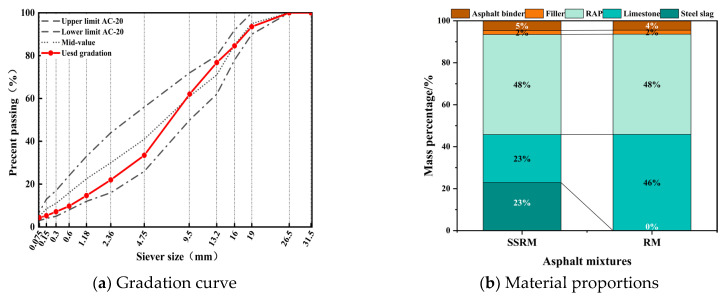
Material proportions and gradation curve of the mixture.

This study employed the Marshall method to design the asphalt mixture proportions (AC-20) with a nominal maximum aggregate size of 19 mm (Figure 3a). Both the Steel Slag–RAP Recycled Mixture (SSRM) and the Limestone–RAP Recycled Mixture (RM) incorporated 50% RAP, which was selected based on engineering practice to achieve high RAP utilization while maintaining acceptable mechanical performance, and their optimal asphalt contents were 4.85% for SSRM and 4.67% for RM, respectively, as shown in Figure 3b. The slightly higher asphalt content in SSRM is attributed to the rough and porous surface characteristics of steel slag aggregates, which increase asphalt absorption. The Marshall test results (Table 5) show that SSRM exhibits slightly higher stability (15.06 kN) and stability/flow ratio (4.81) than RM, indicating improved resistance to permanent deformation while maintaining appropriate volumetric properties. Furthermore, in SSRM, 23% of the coarse aggregate portion was replaced with steel slag, substituting limestone. To comprehensively evaluate the performance differences between SSRM and RM in road engineering, this study tested and analyzed the key road performance characteristics of both mixtures according to the “Test Methods of Bitumen and Bituminous Mixtures for Highway Engineering” (JTG 3410-2025) [15]. Water stability was assessed by the Tensile Strength Ratio (TSR) to evaluate resistance to water damage. High-temperature deformation resistance was determined using the rutting test. Low-temperature cracking resistance was characterized by toughness and fracture properties through the semi-circular bending test. The detailed test conditions and specimen size for all pavement performance tests are presented in Table 6.

### 2.3. Life Cycle Assessment Methodology

To evaluate the environmental performance of asphalt mixtures, this study conducted a “Cradle-to-Gate” Life Cycle Assessment (LCA) in accordance with ISO 14040/14044 standards [16]. The scope of the assessment specifically covered three stages: raw material production, transportation, and construction. Given the high uncertainty in the road use, maintenance, and end-of-life stages, which are significantly influenced by factors such as traffic load, climate conditions, and maintenance strategies, these stages were not included in the system boundary of this study.

1. LCA Framework

The goal of this study is to quantitatively compare the differences in greenhouse gas emissions during the material production, transportation, and construction stages for Steel Slag–RAP Synergy Hot-Recycled Asphalt Mixture (SSRM), traditional Limestone–RAP Mixture (RM), and Virgin Asphalt Mixture (Virgin Mix). The Functional Unit is defined as: “Paving a 2 km long, 10.1 m wide, 7 cm thick, and 4% void ratio single-direction three-lane AC-20 asphalt pavement middle layer on the G30 Lianyungang-Horgos Expressway expansion project section 11 in Xinjiang”.

2. Life Cycle Inventory Analysis

An inventory database was established following the principle of mass-energy balance for life cycle data.

Emission Factors: Primarily sourced from the IPCC National Greenhouse Gas Inventory Guidelines, combined with the IPCC AR6 (2021) greenhouse gas equivalent coefficients [17]. Energy consumption from coal, diesel, gasoline, natural gas, and electricity is all converted into CO_2_ equivalent values. The electricity emission factor is taken as the regional grid baseline value of 0.6 kgCO_2_-eq/kWh.

Activity Data: Data such as material consumption, transportation distances, and machinery fuel consumption are all derived from on-site project surveys, the “Highway Engineering Machinery Unit Cost Quota” (JTG/T3832-2018), and the “Highway Engineering Budget Quota” (JTG/T3833-2018) [18,19].

Allocation Rules: For steel slag and RAP (reclaimed asphalt pavement), this study adopted the Cut-off Method. Since steel slag is a byproduct of steel production and RAP is a waste recycled material, neither is allocated emissions from their original production processes (steelmaking or initial road construction). Only emissions from their subsequent processing (e.g., milling, crushing, screening, preheating) and transportation stages are calculated.

3. Life Cycle Impact Assessment

This study selected the 100-year Global Warming Potential (GWP100) as the environmental impact assessment indicator to measure the total carbon dioxide equivalent emissions within the system boundary. The final results will be aggregated by material, transportation, and construction stages and normalized to the defined functional unit.

4. Sensitivity and Uncertainty Analysis

To verify the robustness of the model results, this study applied appropriate perturbations to key parameters (transportation distance, asphalt content, diesel consumption, material emission factors) based on actual engineering conditions. If the change in GWP100 is controlled within ±10%, the model conclusions are considered to have good robustness.

### 2.4. Economic Benefit Analysis Model

This study establishes an economic analysis framework, aligned with our environmental assessment, to evaluate the financial viability of SSRM compared to RM and Virgin Mix. The analysis scope is “Cradle-to-Gate”, encompassing raw material production, transportation, and construction, consistent with our environmental boundaries. We exclude the in-use, maintenance, and end-of-life phases due to their high variability and dependence on external factors.

1. Goal and Scope

Our objective is to quantify the initial construction costs for both mixtures under an identical functional unit. Cost data are derived from on-site project surveys, regional market prices, and the “Highway Engineering Budget Quota” [19], all standardized to Chinese Yuan (CNY). All cost data were normalized to the 2025 price level, and the cost calculation includes direct material and transportation costs, while taxes and other indirect charges were excluded to ensure consistency and comparability of the economic evaluation. The economic system boundary precisely matches the environmental model to ensure direct comparability.

2. Cost Structure and Calculation

The total cost (M_s_) for each mixture system comprises three distinct components, as illustrated in Equation (1):

(1) $$\begin{array}{c} M_{s}=M_{1}+M_{2}+M_{3} \end{array}$$

where M_1_ represents the direct cost of raw materials (asphalt, aggregates, RAP, mineral powder). M_2_ is the transportation cost, calculated by multiplying the unit freight rate, transportation distance, and material mass. M_3_ covers construction costs, including expenses for mechanical operations such as mixing, loading, paving, and compaction. The costs for each stage are aggregated using Equation (2):

(2) $$\begin{array}{c} M_{i}=\sum_{i=0}^{n}\left(Q_{j,i}\times C_{j,i}\right) \end{array}$$

where Q_j,i_ denotes the number of machine shifts required for process j in stage i. C_j,i_ represents the corresponding unit price or the monetary cost converted from fuel consumption.

3. Transportation Distance-Based Comparison Model

To investigate how sourcing distance impacts overall cost, a linear regression model was developed, using Equation (3):

(3) $$\begin{array}{c} M_{s}=a+b\times D \end{array}$$

where a signifies the baseline cost when transportation distance is zero (representing production stage costs). b denotes the transportation cost intensity per unit distance (CNY/km). D is the transportation distance in kilometers (km). The intersection point of the cost-distance curves for the two mixtures defines the equivalent cost threshold (D_e_q).

## 3. Case Study

### 3.1. Case Description

**Figure 4:**
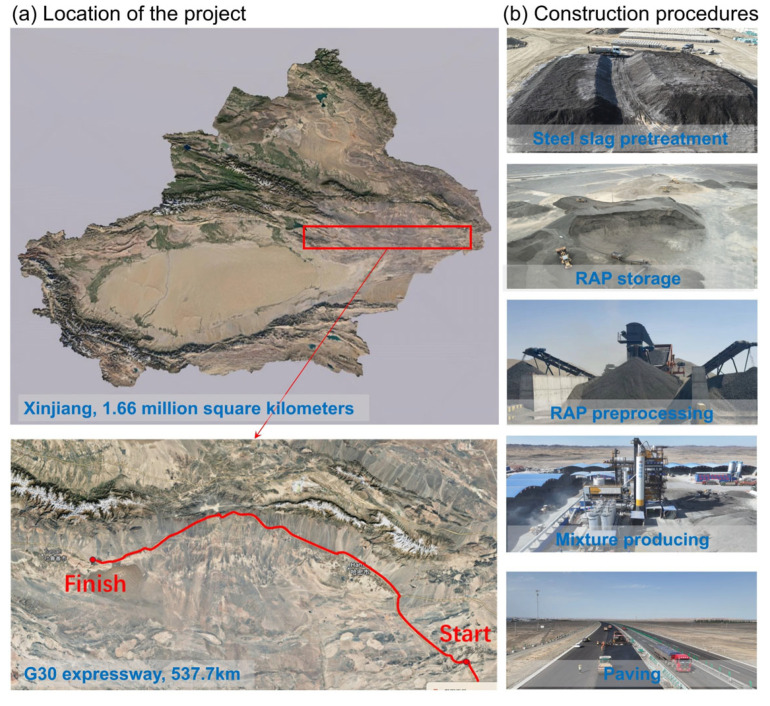
Location and investigation scope of the case study.

The Xingxingxia–Tuyugou section of the G30 Lianyungang–Khorgas Expressway (hereinafter referred to as the “G30 Project”) is located in northern Xinjiang and serves as a vital national transportation corridor connecting the Central Plains with Northwest China, as shown in Figure 4a. The route spans approximately 537.1 km, with an average daily traffic volume exceeding 12,000 vehicles. Situated along the northern edge of the Kumtag Desert, the project area is characterized by Gobi desert and denuded hilly terrain, with elevations ranging from 800 to 1500 m. The region exhibits typical arid zone climatic features in Northwest China, including intense aeolian activity, evaporation far exceeding precipitation, and pronounced diurnal temperature variations. These environmental conditions impose stringent requirements on the high-temperature stability, resistance to aeolian abrasion, and moisture damage susceptibility of the pavement.

In recent years, with the continued growth of regional economic and logistics activities, the service level of the existing dual four-lane expressway has approached saturation. Accordingly, reconstruction and expansion of the G30 Project commenced in 2024, upgrading the original dual four-lane configuration to a dual eight-lane cross-section. The construction of the four new lanes necessitates the milling of the original hard shoulders (emergency lanes) on one side (or both sides in certain sections), generating approximately 1.7 million tons of reclaimed asphalt pavement (RAP). To improve RAP utilization and reduce construction costs, the lower layer of the newly constructed four lanes was designed using plant-mixed hot recycled asphalt mixture (AC-20). In parallel, the G30 Project was selected as a 2024 Technology Demonstration Initiative by the Ministry of Transport of China, featuring the demonstration application of steel slag pavement technology.

This engineering context provides a robust practical platform for the present study. All field data utilized in this research are derived from actual project investigations, ensuring strong engineering representativeness and scientific reference value, as presented in Figure 4b.

### 3.2. Goal and Scope Definition

**Figure 5:**
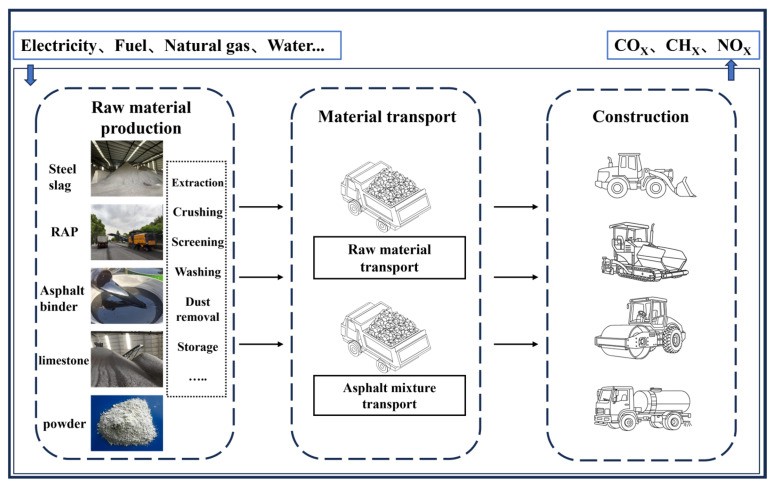
System boundary. **Note:** The scope is limited to “cradle-to-gate”, encompassing raw material production, transportation, and construction stages, while excluding the use, maintenance, and end-of-life phases of the pavement.

To ensure the comparability of the results, the functional unit was defined as: “Assuming the construction of a 2 km-long, 7 cm-thick, and 10.1 m-wide unidirectional three-lane AC-20 asphalt pavement section (with a design air void content of 4%) in Lot 11 of the G30 Expansion Project. This functional unit refers only to the intermediate pavement layer, excluding ancillary facilities such as curbs and markings, as well as subsequent maintenance and rehabilitation activities”. The definition is based on the Chinese Technical Standard of Highway Engineering [20] issued by the Ministry of Transport of China. The air void content of the three comparative pavement scenarios was uniformly controlled at 4% to eliminate interference from structural differences. The system boundary is illustrated in Figure 5.

It should be noted that asphalt mixture is a processed product, and its mixing process is typically categorized under the construction phase. Accordingly, this study incorporates the mixing production stage into the construction phase for unified accounting, rather than listing it separately as material production (in accordance with the ISO 14040 framework [16]).

### 3.3. Life Cycle Inventory (LCI) Analysis

In the study of environmental emissions, life cycle inventory analysis serves as a core component in the assessment of asphalt pavements. Its objective is to quantify the inputs and outputs associated with the functional unit across all stages within the defined system boundary. Inputs primarily include raw materials and energy consumption, while outputs are characterized by greenhouse gas emissions and other relevant environmental burdens. The acquisition and processing of inventory data are critical, as data quality directly determines the scientific validity and reliability of the evaluation outcomes. To ensure comprehensive coverage of all unit processes within the system boundary, this study integrates on-site investigation data from Lot 11 of the G30 Expressway, supplemented by a review of literature and industry statistical reports. Multi-source data were cross-verified and subjected to reasonableness checks to enhance both accuracy and representativeness. The final life cycle inventory data were derived from field surveys, authoritative literature, and relevant standards.

#### 3.3.1. Raw Material Production Stage

This study follows the “cut-off” principle within the Life Cycle Assessment (LCA) framework, including only those typical processes directly associated with raw material extraction and primary processing within the system boundary. This approach ensures data availability and model operability. Within the defined functional unit, the quantities of material inputs are summarized in Table 7. The corresponding energy consumption and emission factors are detailed in Table 8. The sources of emission factors for asphalt materials are compiled in Table 9, while the emission factors for coal, diesel, and natural gas are derived from the IPCC Guidelines for National Greenhouse Gas Inventories [17].

#### 3.3.2. Transportation Stage

The life cycle inventory data for the transportation phase primarily include the hauling distances, transport modes, and characteristic parameters of the delivery vehicles for each material type. Transport distances were determined based on field investigations conducted at Lot 11 of the G30 Project. The primary vehicle used is the 15 t dump truck, and its fuel consumption parameters and emission factors were established in accordance with the Highway Engineering Budget Quota [19] and the IPCC Guidelines for National Greenhouse Gas Inventories [17], respectively. A summary of the assigned transport distances for each material is presented in Table 10. These data serve as fundamental input parameters for subsequent quantitative environmental impact assessments.

#### 3.3.3. Construction Stage

The life cycle inventory data for the construction phase primarily cover the processes of mixing, loading, transport, paving, and compaction, with the associated environmental impacts arising mainly from fuel consumption by construction machinery. Equipment types and machine-shift parameters were determined based on the construction organization plan. Asphalt mixture production was carried out using a continuous mixing plant with a rated capacity not exceeding 320 t/h. Loading and transfer operations of aggregates and mixtures involved the use of wheel loaders and 5 t dump trucks. Paving and compaction were performed by an asphalt paver, a double-drum vibratory roller, and a pneumatic-tire roller, respectively.

## 4. Results and Discussion

### 4.1. Asphalt Mixture Performance

#### 4.1.1. Temperature Sensitivity

**Figure 6:**
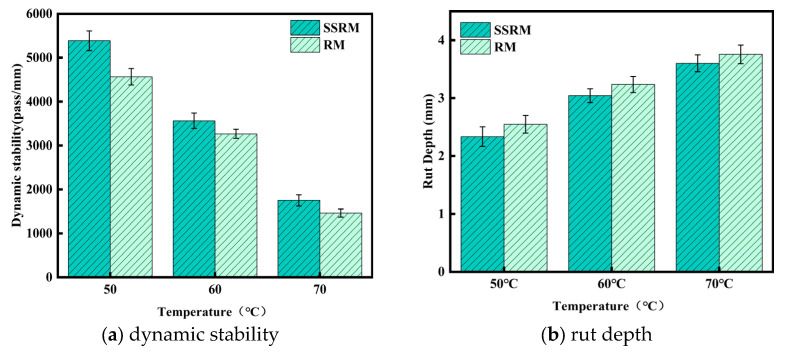
High-temperature performance of asphalt mixtures.

Figure 6 presents a comparison of the rutting test results for the two recycled asphalt mixtures. Within the temperature range of 50 °C to 70 °C, the dynamic stability (DS) decreased as temperature increased, accompanied by a corresponding increase in rut depth. Compared with the conventional recycled mixture (RM), the steel slag–RAP recycled mixture (SSRM) showed higher dynamic stability and lower rut depth across the entire temperature range. The difference between the two mixtures was more noticeable at 50 °C and 60 °C, while the gap became smaller at 70 °C, although SSRM still exhibited slightly better performance. On average, SSRM showed approximately 14–16% higher dynamic stability and about 5–8% lower rut depth compared with RM. These results suggest that the incorporation of steel slag may contribute to improved rutting resistance of the recycled asphalt mixture at elevated temperatures.

**Figure 7:**
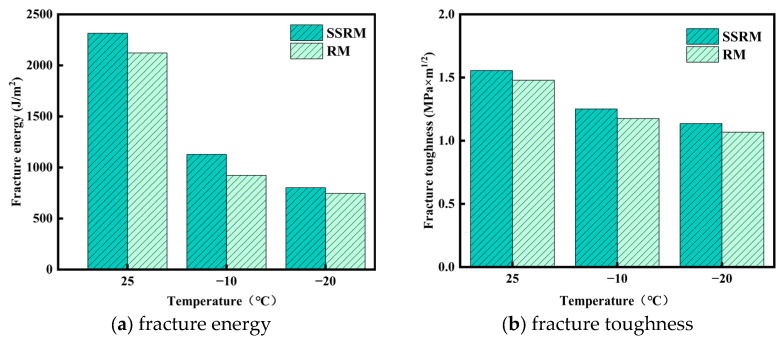
Low-temperature performance of asphalt mixtures.

Figure 7 presents the fracture energy (Gf) and fracture toughness (KIC) of the two mixtures determined by the semi-circular bending (SCB) test. At test temperatures of 25 °C, −10 °C, and −20 °C, the SSRM exhibited higher Gf and KIC values than the RM. At 25 °C, the Gf and KIC of SSRM were approximately 8–12% and 3–5% higher than those of RM, respectively. This advantage became more pronounced at −10 °C, with increases of 20–25% for Gf and 7–10% for KIC. At −20 °C, although the improvement margin narrowed, SSRM still maintained 5–10% and 3–5% higher values for Gf and KIC, respectively. These results indicate that SSRM exhibits superior fracture resistance and energy dissipation capacity under intermediate and low temperature conditions.

The enhanced low-temperature cracking resistance is attributed to the rough and porous surface of steel slag aggregates and their alkaline characteristics, which strengthen asphalt–aggregate adhesion and aggregate interlocking. In addition, the higher asphalt absorption of steel slag leads to a slightly higher optimum asphalt content, resulting in a thicker asphalt film that improves mixture ductility at low temperatures. The aged binder in RAP increases mixture stiffness, while the improved binder–aggregate interaction provided by steel slag helps maintain higher fracture energy and toughness under low-temperature loading.

#### 4.1.2. Moisture Stability

**Figure 8:**
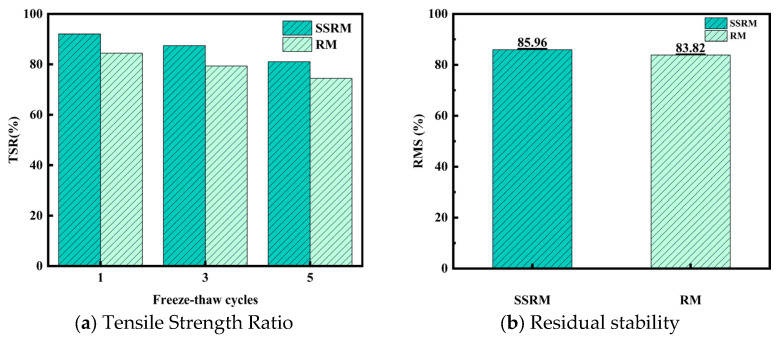
Moisture stability performance of asphalt mixtures.

Figure 8 presents the tensile strength ratio (TSR) of the two mixtures under increasing freeze–thaw cycles. As the number of cycles increased from 1 to 5, the TSR values exhibited a continuous decline, indicating cumulative damage to moisture resistance caused by freeze–thaw action. At each test interval, the SSRM demonstrated higher TSR values than the RM. After one freeze–thaw cycle, the TSR of SSRM was approximately 5–7% higher than that of RM; after three cycles, the improvement ranged from 5% to 6%; and after five cycles, the SSRM still maintained a 6–8% advantage. The results of the immersed Marshall test further supported this trend: the retained stability of SSRM was 85.96%, compared to 83.82% for RM, reflecting an absolute increase of approximately 2.14% and a relative improvement of about 2.6%. These findings demonstrate that under coupled freeze–thaw and moisture exposure, the SSRM exhibits superior resistance to stripping and better retention of mechanical strength, indicating enhanced moisture stability and durability relative to the RM.

#### 4.1.3. Skid Resistance

**Figure 9:**
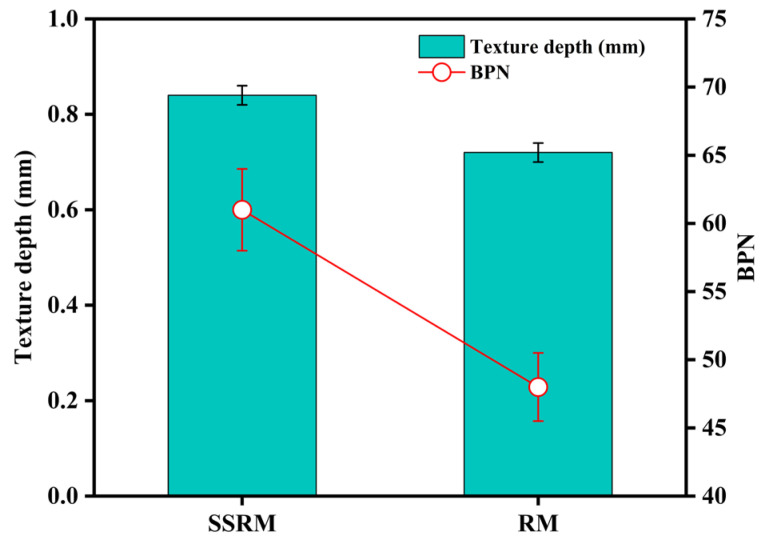
Texture depth and BPN of SSRM and RM.

Figure 9 compares the skid resistance parameters of the two mixtures. The texture depth of SSRM is approximately 0.83 mm, notably higher than that of RM (0.72 mm), representing an increase of about 15%. Its British Pendulum Number (BPN) is approximately 60, compared to 49 for RM, corresponding to an improvement of around 22%. These results indicate that SSRM exhibits superior skid resistance performance. This advantage can be attributed to two primary factors. First, the greater macro-texture depth of SSRM facilitates water film dissipation and enhances drainage at the tire–pavement interface, thereby improving macro-scale shear resistance. Second, the rough and angular surface characteristics of steel slag aggregates contribute to increased micro-scale friction components, leading to higher effective friction at the contact interface. The combined effect of these mechanisms endows SSRM with enhanced surface adhesion and prolonged skid resistance under wet conditions, which is beneficial for mitigating hydroplaning risks and improving traffic safety.

### 4.2. Environmental Impact Analysis

#### 4.2.1. Emissions Analysis by Material

**Figure 10:**
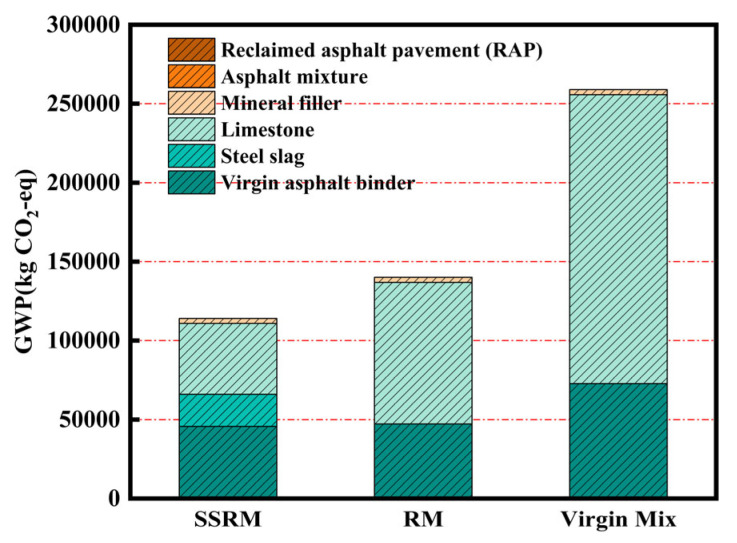
GWP100 of the raw material production phase.

Figure 10 and Table 11 present a comparison of the 100-year global warming potential emissions from the raw material production phase of the three mixtures. Both SSRM and RM exhibited substantially lower total emissions than the virgin mixture, with SSRM showing the lowest value among all. These results demonstrate that, under the same functional unit, the use of recycled materials and industrial by-products in place of virgin resources can effectively reduce the environmental burden of pavement materials. In terms of material contribution, virgin asphalt binder was the predominant source of greenhouse gas emissions, accounting for over one-quarter of the total emissions for each mixture. Its production process is energy-intensive and characterized by high emission factors, making asphalt binder content a key variable influencing the overall environmental performance.

Notable differences were observed in the contribution from aggregates. The limestone aggregate used in RM generates emissions primarily from quarrying, crushing, and long-distance transportation. In contrast, steel slag—used in SSRM as a metallurgical by-product—carries a substantially lower carbon footprint when allocated using the cut-off method, under which upstream environmental loads are attributed to the primary production system, leaving the road system with only marginal burdens. Furthermore, compared to the virgin mixture, both SSRM and RM reduce the demand for virgin aggregate and virgin asphalt binder through the incorporation of RAP. SSRM further replaces a portion of the remaining virgin aggregate with steel slag, achieving a dual-emission reduction effect through the combined use of RAP and steel slag. Collectively, these findings highlight the significant environmental benefits of using steel slag and RAP, providing empirical support for the design of low-carbon pavement materials and for engineering practices aligned with carbon neutrality goals.

**Figure 11:**
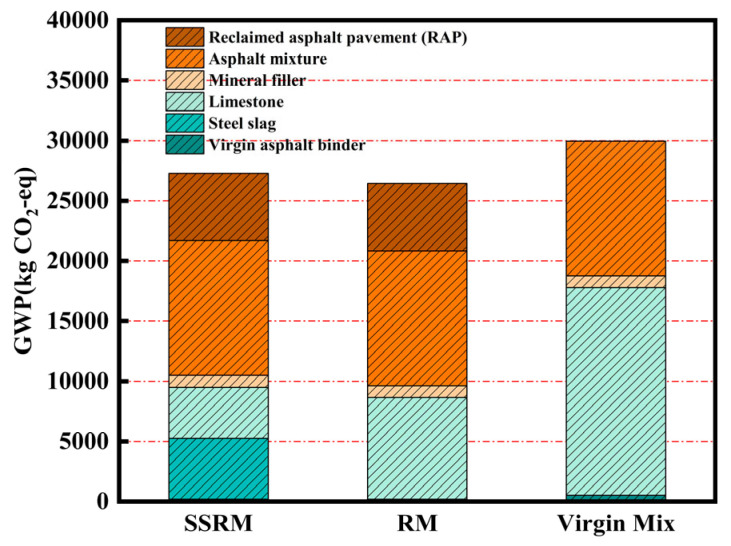
GWP100 of the transportation phase.

Figure 11 and Table 12 and Table 13 present a comparison of the 100-year global warming potential (GWP100) emissions for the three mixtures during the transportation and construction phases. Overall, the Virgin Mix exhibited the highest total emissions, while both SSRM and RM showed lower values than the Virgin Mix, with SSRM slightly higher than RM. During the transportation phase, the hauling of RAP and recycled asphalt mixtures was the primary contributor to GWP. Emissions from the transport of steel slag exceeded those from limestone aggregate, primarily due to the higher density of steel slag, which results in greater fuel consumption and associated carbon emissions when transporting an equivalent volume of material. Consequently, replacing natural aggregate with steel slag introduces a certain increase in greenhouse gas emissions during the transportation stage.

Although the GWP of SSRM during transportation was marginally higher than that of RM, it remained lower than that of the Virgin Mix, retaining a modest advantage. When considered alongside the substantial emission reductions achieved during the raw material production phase, the additional transportation-related emissions associated with steel slag do not offset the environmental benefits gained from resource substitution and waste valorization. Overall, SSRM still offers superior comprehensive environmental performance.

#### 4.2.2. Emissions Analysis by Stage

**Figure 12:**
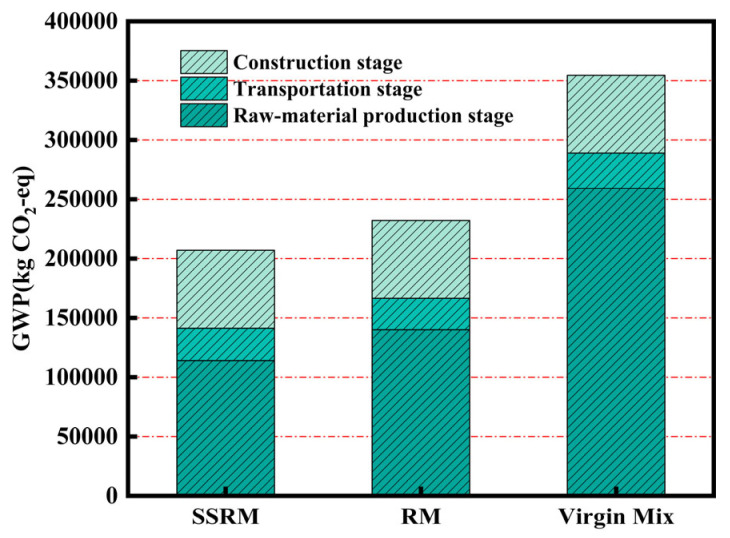
Comparison of cradle-to-gate GWP100 across all stages.

Figure 12 compares the life-cycle greenhouse gas emissions of the three asphalt mixtures. SSRM exhibited the lowest total emissions, followed by RM, while the Virgin Mix showed the highest value. The three mixtures presented comparable emission levels during the construction and transportation phases, with the primary differences observed in the raw material production stage. Specifically, the total emissions of SSRM were approximately 10.84% lower than those of RM, and 41.62% lower than those of the Virgin Mix. RM showed a reduction of about 34.52% compared to the Virgin Mix. These results demonstrate that SSRM offers substantial advantages in reducing greenhouse gas emissions, particularly when compared to the Virgin Mix.

This environmental benefit is largely attributed to the synergistic use of steel slag and RAP. Without compromising pavement performance, SSRM effectively reduces the carbon footprint of the raw material production phase by substituting natural resources with industrial by-products and recycled materials. This approach represents a replicable technical pathway toward low-carbon construction and resource circularity in road engineering.

#### 4.2.3. Effect of Transport Distance on GWP and Cost

**Figure 13:**
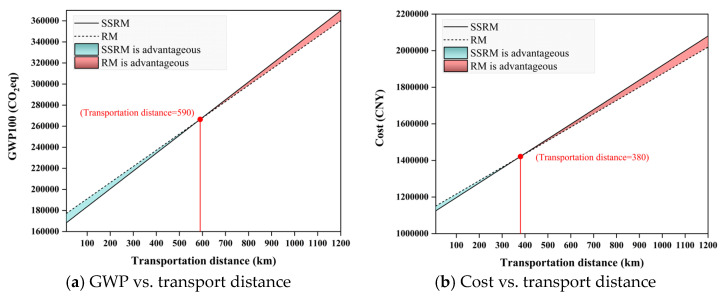
Comparison of GWP and Cost under different transport distances.

Figure 13a presents a comparison of the cumulative GWP100 emissions of SSRM and RM as a function of transport distance. The intercept represents carbon emissions from the raw material production phase, while the slope reflects the emission intensity per unit transport distance. SSRM exhibits notably lower production-stage emissions than RM, albeit with a slightly higher emission intensity per kilometer of transport. The two curves intersect at approximately 590 km, defining an emission equivalence threshold. When the transport distance is below 590 km (blue-shaded region), SSRM yields lower total emissions than RM. Beyond this distance (red-shaded region), RM becomes the lower-emission option. In practical engineering projects, the transport distance of aggregates used in road construction is typically within several tens of kilometers, depending on the availability of local material sources. In this study, larger transport distances (e.g., up to several hundred kilometers) were also considered as sensitivity-analysis scenarios. Within the analyzed range, SSRM generally shows lower total carbon emissions than RM.

Figure 13b illustrates the total cost of SSRM and RM as a function of transport distance. Both fitted curves exhibit an approximately linear increase, with the intercept representing the baseline cost at the production stage and the slope reflecting the cost increment per unit transport distance. SSRM shows a lower production cost than RM, but a slightly higher transport cost per kilometer. The two curves intersect at approximately 380 km, defining a cost equivalence threshold. When the transport distance is less than 380 km (blue-shaded region), SSRM achieves a lower total cost than RM. Beyond this distance (red-shaded region), RM becomes more cost-competitive. In practice, the typical hauling radius for aggregate materials in road construction ranges from 300 to 400 km. Within this range, SSRM maintains a cost advantage under most conditions. These findings indicate that SSRM not only delivers carbon emission reductions but also offers favorable economic feasibility within reasonable transport distances.

**Figure 14:**
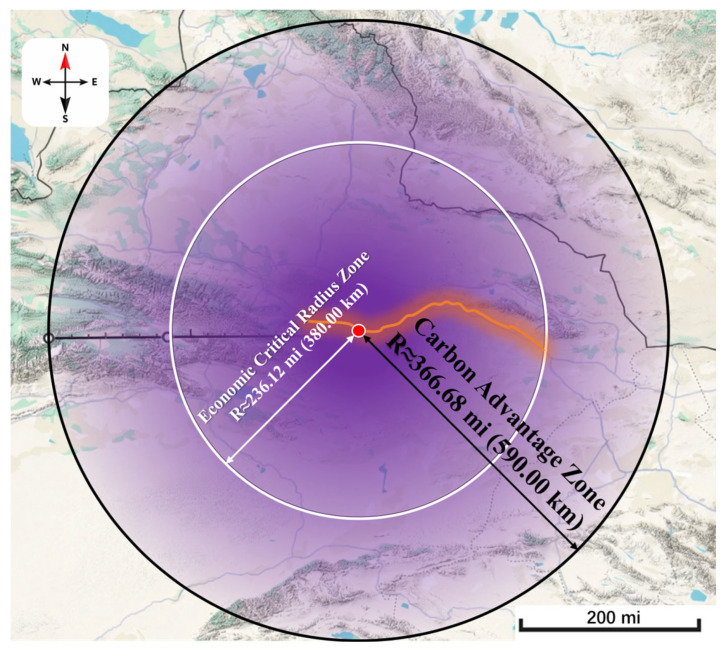
Schematic diagram of the environmental and economic advantages of steel slag recycling.

To provide spatial decision support for construction projects (e.g., site selection, technology choice, and transport radius), this study takes the project site (red dot) as the center and conducts a comprehensive analysis using the carbon emission and cost model under the “cradle-to-gate” boundary. The results are shown in Figure 14.

The white circle (radius ≈ 380 km) represents the economic critical radius. Within this range, the total cost of SSRM is lower than that of RM. The black circle (radius ≈ 590 km) indicates the carbon emission advantage zone, where the GWP100 of SSRM is lower than that of RM. The purple gradient band illustrates the gradual decline in the comprehensive advantage of SSRM as transport distance increases. The deep purple area at the center indicates the strongest dual advantage in both economic performance and carbon emissions. Inside the white circle is the dual-advantage zone, where SSRM offers both economic and environmental benefits. The area between the white and black circles represents the carbon-advantage-only zone, where SSRM still reduces emissions but is slightly less economically competitive.

### 4.3. Economic Benefit Analysis

**Figure 15:**
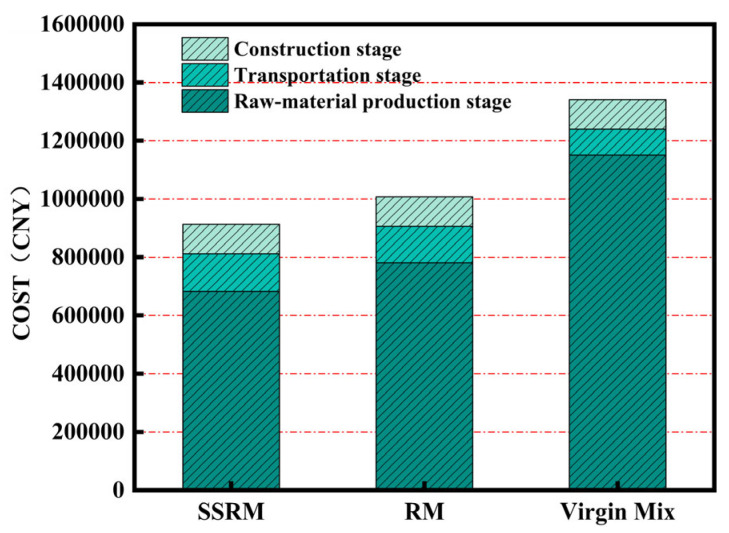
Comparison of costs between SSRM and RM (CNY).

The cost data for SSRM, RM, and Virgin Mix are presented in Figure 15 and Table 14 and Table 15. Compared to RM and Virgin Mix, the cost of SSRM is reduced by approximately 9.37% and 34.51%, respectively. This indicates that substituting natural limestone with steel slag combined with the use of RAP can effectively lower material costs. In comparison, the total cost of RM is about 27.74% lower than that of Virgin Mix. Although RM also incorporates RAP, its overall cost remains significantly higher than that of SSRM due to the relatively high cost of limestone aggregate. In summary, SSRM shows the most favorable cost performance across all evaluated scenarios. It effectively reduces the direct cost of road construction and offers a viable alternative for material selection in pavement engineering.

### 4.4. Sensitivity Analysis

**Figure 16:**
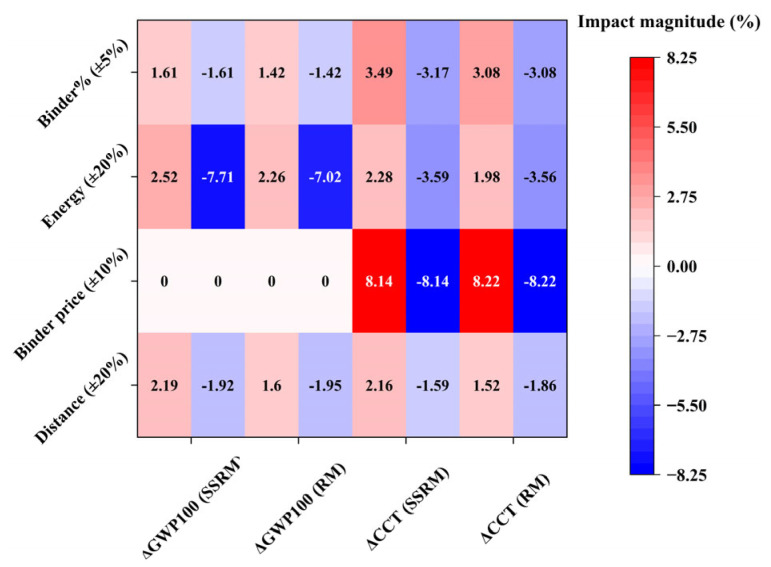
Uncertainty analysis of key factors.

A sensitivity analysis was conducted to evaluate the influence of four factors—asphalt content, energy consumption, asphalt price, and transport distance—on GWP and cost. The variation ranges were defined based on engineering practice and literature data: asphalt content ±5%, asphalt price ±10%, energy consumption ±20%, and transport distance ±20%. The results are presented in Figure 16. The four factors exhibit notably different magnitudes of impact on cost (ΔCCT) and environmental impact (ΔGWP100). In the figure, red indicates a positive amplifying effect, blue indicates a negative mitigating effect, and color intensity increases with sensitivity strength. In terms of cost, the most sensitive factor is asphalt price. A ±10% variation in asphalt price results in a ΔCCT ranging from +8.22% to –8.14%, which is substantially higher than the effects of the other factors. Regarding environmental impact, the dominant factor is energy consumption. A ±20% variation in energy consumption leads to a ΔGWP100 ranging from –7.71% to +2.52%.

## 5. Conclusions

(1) The steel slag–RAP recycled asphalt mixture (SSRM) exhibits superior overall performance compared with the limestone–RAP recycled asphalt mixture (RM) in terms of high-temperature rutting resistance, low-temperature cracking resistance, moisture stability, and skid resistance. Specifically, SSRM shows approximately 14–16% higher dynamic stability and 20–25% higher fracture energy at −10 °C, indicating improved resistance to rutting and cracking. The test results indicate that the performance indicators of SSRM meet the relevant specification requirements (JTG 3410-2025) [15]. This performance improvement is mainly attributed to the high strength of steel slag aggregates and their rough, porous surface with alkaline activity, which enhances asphalt–aggregate adhesion and aggregate interlocking. These findings confirm the excellent engineering feasibility of utilizing steel slag as a replacement for natural limestone coarse aggregates.

(2) The greenhouse gas emissions of SSRM are lower than those of RM and the virgin asphalt mixture (Virgin Mix). Specifically, the life-cycle global warming potential (GWP) of SSRM is approximately 10–11% and 40% lower than that of RM and Virgin Mix, respectively. The raw material production phase contributes the most to the life-cycle GWP, with virgin asphalt consistently being the primary carbon source. Furthermore, when the transportation distance for steel slag is less than 590 km, the total carbon emissions of SSRM remain lower than those of RM. Consequently, within a reasonable radius for local material sourcing, the steel slag-RAP synergistic system not only offers performance advantages but also significantly reduces the carbon footprint during the construction of intermediate pavement layers.

(3) The initial construction cost of SSRM is approximately 9–10% and 34–35% lower than that of RM and Virgin Mix, respectively. This cost reduction is mainly due to the lower unit price of steel slag and the substitution of virgin materials with RAP. However, transportation cost plays a critical role in determining the overall economic feasibility of SSRM. As the transportation distance increases, the cost advantage gradually decreases because transportation constitutes a significant component of the total production cost of asphalt mixtures. SSRM demonstrates a distinct cost advantage when the transportation distance is within 380 km.

In conclusion, considering pavement performance, emissions, and initial construction costs, the steel slag–RAP recycled asphalt mixture demonstrates superior comprehensive performance compared to traditional limestone–RAP recycled mixtures and virgin mixtures. It qualifies as a green pavement material with both engineering feasibility and sustainability. The evaluation and decision-making framework proposed in this paper, centered on the concepts of “local material sourcing radius–emission parity threshold–cost parity threshold”, can provide a quantitative basis for material selection, plant location, and transportation logistics in similar engineering projects. This framework offers valuable references for promoting the engineering application of the steel slag-RAP synergistic system and for formulating relevant policies. The scope of this study remains limited, and key parameters related to pavement structural design, such as resilient modulus and fatigue resistance, were not considered. Further research is needed to evaluate the long-term durability and structural applicability of steel slag–RAP recycled mixtures.

## Figures and Tables

**Table 1:** Basic Properties of Steel Slag.

Indicator	5–10 mm	10–16 mm	Requirements
Apparent Relative Density	3.469	3.230	≥2.6
Bulk Relative Density	3.029	3.121	-
Water Absorption/%	2.18	1.08	≤3
f-CaO Content/%	0.31	0.18	≤3
Crushing Value/%	12.4		≤22.0
Bitumen Adhesion Grade	5		≥4
Los Angeles Abrasion/%	16.8		≤28.0

**Table 2:** Basic Properties of Limestone.

Test Indicator	0–5 mm	5–10 mm	10–20 mm	Requirements
Apparent Relative Density	2.745	2.775	2.750	≥2.6
Bulk Relative Density	2.710	2.689	2.675	-
Water Absorption/%	0.81	0.69	0.47	≤3.0
Crushing Value/%	15.9			≤26
Bitumen Adhesion Grade	4			≥4
Los Angeles Abrasion/%	22			≤28

**Table 3:** RAP Analysis Results.

RAP Size	Passing Percentages Through the Following Sieve Sieves										
	19	16	13.2	9.5	4.75	2.36	1.18	0.6	0.3	0.15	0.075
11–22 mm	92.5	79.6	57.9	29.1	18.6	14.1	11.4	8.8	6.2	4.8	3.1
8–11 mm	100	100	99.8	77.9	24.4	18.2	14.1	10.7	7.9	6.4	4.9
0–8 mm	100	100	100	97.0	54.1	32.6	23.6	17.0	12.1	9.6	6.8

**Table 4:** Asphalt Analysis Results.

Indicator	Test Results	Test Method
Penetration (25 °C, 100 g, 5 s)/0.1 mm	65.0	T 0604-2011
Ductility (15 °C)/cm	>150	T 0605-2011
Softening point (Ring-and-Ball)/°C	48.0	T 0606-2011
Flash point/°C	>270	T 0611-2011
Density (15 °C), g/cm^3^	1.035	T 0603-2011

**Table 5:** Marshall test results.

Mixture Type	Asphalt Content (%)	Bulk Density (g/cm^3^)	Theoretical Maximum Density (g/cm^3^)	Stability (kN)	Flow (mm)	Stability/Flow	Va (%)	VMA (%)	VFA (%)
SSRM	4.85	2.47	2.572	15.06	3.13	4.81	4.07	12.91	68.49
RM	4.67	2.44	2.541	14.27	3.08	4.63	3.91	12.71	69.23

**Table 6:** Specimen size and main test conditions.

Property	Specimen Size	Main Test Conditions
High-temperature deformation resistance	300 mm × 300 mm × 50 mm	Test temperatures of 50 °C, 60 °C and 70 °C, wheel load of 0.7 MPa, wheel with moving speed of 42 times/min
Low-temperature crack resistance	φ148 mm × 50 mm (semi-circular specimen)	Environmental chamber of −25 °C, −10 °C, 20 °C, specimen deformation rate of 0.5 mm/min
Moisture stability	φ101.6 mm × 63.5 mm	Water bath of 60 °C, freezer of −18 °C
skid resistance	300 mm × 300 mm × 50 mm	Texture depth measured by the sand patch method; BPN measured using a British Pendulum Tester at room temperature (20 ± 5 °C).

**Table 7:** Mass of raw materials in asphalt mixtures.

Asphalt Mixture	Steel Slag	Limestone	RAP	Mineral Filler	Added Virgin Asphalt
AC-20 (Limestone)	0 t	1534 t	1697 t	63.9 t	99.1 t
AC-20 (Steel slag)	765.3 t	765.4 t	1697 t	64.1 t	102.5 t

**Table 8:** Greenhouse gas emission factors by energy type.

Energy Type		Coal (kg)	Gasoline (kg)	Diesel (kg)	Heavy Fuel Oil (kg)	Natural Gas (m^3^)	Electricity (kW·h)
Default emission factor (kg/MJ)	CO_2_	0.094600	0.069300	0.074100	0.077400	0.056100	0
	CH_4_	0.000001	0.000003	0.000003	0.000003	0.000001	0
	N_2_O	0.0000015	0.0000006	0.0000006	0.0000006	0.0000001	0

Note: The emission factor for electricity is indicated as “0” in the table, reflecting that electricity does not directly correspond to combustion emissions. Its emission factor should be determined based on the baseline emission factor of the regional power grid. In this study, a default value of 0.6 kg CO_2_ e/kWh is adopted for electricity, referencing the IPCC National Greenhouse Gas Inventory Guidelines and published data from the Ministry of Ecology and Environment [17].

**Table 9:** Sources of asphalt emission data.

Data Source	Emission Factor (kg CO_2_ e/t)	Remarks
Ecoinvent 3.8 [21]	408–540	Varies depending on production region and energy structure
GaBi database [22]	500	European average
Simapro China customized database [23]	370–470	Simulated values based on Sinopec/CNPC production data
Literature review [4,24,25]	420–480	Life cycle average for base asphalt in China
Average	424.5–497.5 (461)	Average

**Table 10:** Transport distances of raw materials and asphalt mixtures.

Material Type	Transport Distance (km)
Asphalt binder	30
Steel slag	100
Limestone aggregate	100
Mineral filler	30
RAP	30

**Table 11:** Greenhouse gas emission inventory for the raw material production phase.

Material Type	Emission Factor (kg CO_2_e/kg)	SSRM (kg CO_2_e)	RM (kg CO_2_e)	Virgin Mix (kg CO_2_e)
Virgin asphalt binder	0.461	45,685.1	47,252.0	72,755.5
Steel slag	0.100	20,364.0	0	0
Limestone aggregate	0.050	44,800.0	89,601.6	182,936.6
Mineral filler	0.4721	3204.6	3204.6	3024.6
Asphalt mixture	0	0	0	0
Reclaimed asphalt pavement (RAP)	0	0	0	0
Total	–	114,054.5	140,058.7	258,896.7

**Table 12:** Greenhouse gas emission inventory for the transportation phase.

Material Type	Transport Equipment/Dump Truck	Distance (km)	SSRM (kg CO_2_e)	RM (kg CO_2_e)	Virgin Mix (kg CO_2_e)
Virgin asphalt binder	5 t	30	193.112	186.718	520.83
Steel slag	15 t	100	5071.367	0	0
Limestone aggregate	15 t	100	4226.139	8452.278	17,256.73
Mineral filler	15 t	30	993.005	993.005	993
Asphalt mixture	15 t	30	11,200.649	11,200.649	11200.7
Reclaimed asphalt pavement (RAP)	15 t	30	5600.324	5600.324	0
Total	–	–	27,284.596	26,432.973	29,971.22

**Table 13:** Greenhouse gas emission inventory for the construction phases.

Equipment Type	Machine-Shift	SSRM/RM/Virgin Mix (kg CO_2_e)
5 t dump truck (mixing plant transport)	2.064	177.53
Wheel loader (3 m^3^)	3.733	516.34
Asphalt mixing plant (≤320 t/h)	1.739	63,309.71
Asphalt paver (12.5 m)	2.064	610.86
Water truck (10,000 L)	0.707	236.76
Double-drum vibratory roller (≤15 t)	8.682	362.31
Pneumatic-tire roller (16–20 t, intermediate compaction)	2.885	190.12
Pneumatic-tire roller (20–25 t, final compaction)	5.543	226.01
Total	–	65,629.63

**Table 14:** Cost summary by life cycle stage.

Material Type/Stage Cost (CNY)	Raw Material Production Stage		Transportation Stage		Construction Stage	Total Cost	
	SSRM	RM	SSRM	RM	SSRM/ RM	SSRM	RM
Base asphalt	512,500	495,500	916.19	885.85	101,691.9	930,172.92	990,588.56
Steel slag	4072.8	0	24,060.2	0			
Limestone	85,528.8	171,057.6	20,050.1	40,100.3			
Filler	12,082.64	12,082.64	4711.1	4711.1			
RAP	0	0	53,139.4	53,139.4			
Asphalt mixture production	84,850	84,850	26,569.7	26,569.7			
Total	699,034.24	763,490.24	129,446.78	125,406.42	101,691.9		

**Table 15:** Cost summary for Virgin Mix by life cycle stage.

Material Type/Stage Cost (CNY)	Raw Material Production Stage	Transportation Stage	Construction Stage	Total Cost
Base asphalt	789,105	2470.98	101,691.85	1,341,175.63
Steel slag	0	0		
Limestone	349,242.62	81,871.42		
Filler	12,082.64	4711.13		
RAP	0	0		
Asphalt mixture production	0	0		
Total	1,150,430.24	89,053.54		

## Data Availability

The original contributions presented in this study are included in the article. Further inquiries can be directed to the corresponding author.
